# Consumer Choice between Food Safety and Food Quality: The Case of Farm-Raised Atlantic Salmon

**DOI:** 10.3390/foods5020022

**Published:** 2016-03-25

**Authors:** Morteza Haghiri

**Affiliations:** Memorial University—Grenfell Campus, 20 University Drive, Corner Brook, NL A2H 5G4, Canada; mhaghiri@mun.ca; Tel.: +1-709-637-2190; Fax: +1-709-639-8125

**Keywords:** food safety, food quality, HACCP, farm-raised Atlantic salmon

## Abstract

Since the food incidence of polychlorinated biphenyls in farm-raised Atlantic salmon, its market demand has drastically changed as a result of consumers mistrust in both the quality and safety of the product. Policymakers have been trying to find ways to ensure consumers that farm-raised Atlantic salmon is safe. One of the suggested policies is the implementation of integrated traceability methods and quality control systems. This article examines consumer choice between food safety and food quality to purchase certified farm-raised Atlantic salmon, defined as a product that has passed through various stages of traceability systems in the province of Newfoundland and Labrador, Canada.

## 1. Introduction

The Newfoundland and Labrador (NL) fishing and aquaculture industries continued playing an important role in the provincial economy in 2014. The industry contributed nearly CAD 1 billion to the viable economy of both rural and urban areas despite a 13.1 percent reduction in comparison to 2013 due to the higher market prices for some seafood products that offset the decrease in production in 2014 [1]. The NL fishing and aquaculture industries gained from the consistent increase in global demand for seafood products [2]. The industry also provided year-round jobs in the harvesting and fish processing sectors for more than 18,000 people in the province. Moreover, the Newfoundland and Labrador Department of Fisheries and Aquaculture recorded a 25.1 percent growth in the volume of the aquaculture production in 2014 in comparison to 2013 [3]. Since the 2000s, a series of communicable diseases in the agricultural food industry has occurred that has made consumers monitor carefully the quality of foods they purchase. For instance, the food incidence of polychlorinated biphenyls (PCBs) in farmed Atlantic salmon initially reduced a significant amount of global demand for the product [1,4,5]. To regain consumers’ demand, several proposals from farm-gates to food-on-plates were suggested to mitigate consumer mistrust in the quality of farmed Atlantic salmon. Amongst those policies, the integration traceability methods and quality control systems have been the most recommended [2]. These methods comprise a sequence of operations in the food chains that provide opportunities for consumers to have more access to information that can help them choose the right products and improve their confidence in the origin of foods [6]. The integration traceability methods and quality control systems in the aquaculture industry, in principle, consists of three mechanisms: (i) the GlobalGAP and Quality Management Program (QMP); (ii) the Hazard Analysis and Critical Control Points (HACCP); and (iii) the radio frequency identification and quick response code- systems. The first mechanism mandates the aquaculture industry adopt rigorous procedures on the grounds of the second one, *i.e.*, the HACCP mechanism. The radio frequency identification and quick response code-systems is used to monitor carefully in-and-out-house salmon operations, which are comprised of cultivation, inspection, distribution, and retailing in the global market [7]. 

As a result of the above policies, the public has paid much attention to the issues of food safety and food quality and thus demand for foods having these concerns in mind has consistently been increased. This increase in demand for high quality foods, including farmed Atlantic salmon is strengthened by an increase in overall household annual income at each level of income-brackets. Similar to any type of food industry, food safety and food quality are two important aspects in the aquaculture industry. These are two different concepts that collectively have the ability to ensure consumers that the product has met the industry’s standards to be purchased. Food safety refers to the concept that diseases like pathogenic microorganisms, misuse of food additives and contaminants such as chemical or biological toxins and adulteration are prevented, whereas food quality is comprised of a series of consumers’ attributes that collectively influence them to put different values on different products at the time of purchase. Some of these attributes are place of origin, freshness, colour, flavour, texture, taste, nutritional value, the animal welfare, fat content of foods, environmentally friendly production, and sustainable farming practices [8,9]. Although the two concepts have fundamentally different aspects, their collegial meanings make consumers purchase certified foods defined as one that has passed through various stages of the integration traceability methods and quality control systems. For example, if a type of food is contaminated with microorganisms, it can cause health hazards (*i.e.*, food safety perspective) and quickly spread over food products (*i.e.*, food quality).

The main objective of this article is to examine consumer preferences to choose between food safety and food quality at the time of purchasing certified farm-raised Atlantic salmon in the province of Newfoundland and Labrador, Canada. We believe that the integration traceability methods and quality control systems (*i.e.*, QMP, HACCP, and the radio frequency identification and quick response code systems) should seriously be contemplated by the stakeholders in the industry. Thus, it is more appropriate to know now how people think about the implementation of this proposal and how their decisions are being moved (if there is any) towards the concepts of food safety and food quality. We also recommend some policies that can be followed by provincial government to prepare proper infrastructure for the aquaculture industry when the implementation of the policy begins.

The rest of the paper is organized as follows. Section 2 briefly reviews recent studies in which consumer perceptions towards the integrating traceability methods and quality control systems in the agricultural food products are analyzed. Section 3 presents the empirical analysis of this research where it explains summary of data, methods of survey, and provides the results of the regression analysis followed by an in-depth discussion about the findings of the empirical analysis. Section 4 concludes the article by presenting some remarkable points, indicating the limitations of the research and providing new directions for further research.

## 2. Recent Studies on Traceability Systems

Since the early 2000s, few studies have investigated the implementation of traceability systems in the global agriculture food sector. In this section, we briefly review these studies and refer interested readers to find more in Golan *et al.* [10,11], Haghiri [2,6,12,13,14,15], Haghiri and Simchi [16], Hobbs [17,18], and Hobbs *et al.* [19]. 

Roheim *et al.* [20] carried out a joint experimental research to investigate consumer preferences for two types of seafood products (*i.e.*, wild and certified farmed salmon and shrimp) in the State of Rhode Island, USA. The regional aquaculture certification group set the standards for a product to be labelled as a certified product. Roheim *et al.* [20] defined the criteria for a certified product by taking into account the following factors: sustainability of fish feed, the level of antibiotic used, water quality, and stocking density. The researchers collected related information from a sample of 250 households in 2010 and used a logit model to examine their decisions to buy wild or certified farm-raised salmon and shrimp. Roheim *et al.* [20] concluded that consumers would buy both wild and certified salmon and shrimp only if the stated standard criteria were met. The authors also reported that the lack of knowledge about different stages of aquaculture production procedures had a negative impact on consumers’ decisions.

Haghiri and Simchi [16] identified different consumers’ segments that were willing to purchase certified farm-raised Atlantic salmon in the province of Newfoundland and Labrador Canada. The following three inquiries were the core of the researchers’ study: “(i) how do consumers value different aspects related to the establishments of mandatory traceability and labeling systems for the farmed Atlantic salmon; (ii) is there a possibility that consumers are segregated into different homogenous groups according to their socioeconomic characteristics and perceptions of various aspects related to the mandatory traceability and labeling systems for farmed Atlantic salmon?; and (iii) given the findings in the second enquiry, what are the main profiles of each consumer’s segment?” [16]. To find answers, the authors carried out a consumer survey (120 participants) in spring 2010 and collected information related to the demographic, socioeconomic, and psychographic variables. The results showed three different factors that affected respondents’ decisions on purchasing certified farm-raised Atlantic salmon. These three factors were labelled as “costly requirement,” ‘‘beneficial requirement,’’ and ‘‘unnecessary requirement.” The costly requirement factor consisted of respondents with positive personal attitudes towards the implementation of traceability systems and labelling regulations for farm-raised Atlantic salmon. This factor that accounted for 28.4 percent of the total variation showed that the participants in the survey expressed their concerns about the operating costs of traceability systems that could potentially generate conflict of interest between consumers and producers [2]. The beneficiary requirement factor expressed the fact that households indeed gained from the traceability system since it would ensure the quality of the food to the consumers. This factor accounted for 23.15 percent of the total variation. The last factor, *i.e.*, unnecessary requirement that accounted for 19.35 percent of the total variation indicated that traceability systems would not be associated with any more information for consumers, as they already believed in the safety of the product [16].

Mai *et al*. [21] used a cost-benefit analysis approach in the Iceland seafood industry and assessed firms’ net benefits at each stage of production and distribution from implementing traceability systems. The research assumed that both costs and benefits amongst the firms in the sample were unequally distributed. The findings of Mai and coworkers’ study were identifying the sources of disparity that caused an unequal distribution of cost and benefits, including liability claim and lawsuits reduction, market growth, labour savings, market growth, and process improvement. Similar studies identified other factors that could have substantial impact on consumers’ perceptions on the implementation of integrated traceability systems in the global food markets. Among those factors, we can refer to cultural diversities [22], lack of quality assurance methods [23], the use of a non-proper risk management methods [24], and consumer attributes such as health, quality, place of origin and naturalness, safety, animal welfare, and control [12,13,14,15,25,26].

Verbeke and Ward [27] examined consumer preferences in quality, traceability and originality for beef labels in Belgium. The authors broke down consumers’ interest, specified as the “level of perceived importance attached to and attention paid to label cues” on gaining more information from reading beef labels and raise their common knowledge on the quality and originality of the product [27]. The focus of their study was to evaluate the mandatory traceability and product originality methods that were applied to European beef products. The result of the study indicated that: (i) consumers placed great values for acquiring information related to quality guarantee seal and/or expiration date of the product than other attributes such as originality and traceability mechanisms; and (ii) consumers believed that improving their knowledge would directly affect their perceptions on food quality and originality.

Evidence showed that integrated traceability systems in the food supply chains could also emerge from regulatory and industry initiatives [28]. Traceability and liability would make all the stakeholders in the industry better off so that they could examine their rights in due diligence [28]. An experimental auction was designed to assess consumer preferences for credence attributes for pork and beef. A credence good is the one for which consumers can neither ascertain its utility impact nor measure its utility gain or loss, even after consumption. Hobbs [28] showed that certified products were usually recognized and highly valued by consumers, especially if they were bundled with other criteria related to quality assurance information [28]. Similar to the results of other studies, Hobbs [28] concluded that the integrated traceability systems could entice food safety and quality assurance in the food industry [25]. 

Gracia and Zeballos [29] conducted a survey in Zaragoza, Spain to examine producers and consumers’ attitudes on the European Union’s traceability and labelling systems in the beef industry. The researchers classified respondents in the survey on the basis of their attitudes towards mandatory traceability and labelling requirements in Europe. The first group was comprised of those respondents who agreed on the implementation of traceability for beef. On the contrary, the second group consisted of those respondents who believed that the product was safe and therefore traceability methods were not required. The third group included those respondents who were indifferent between one way and the other. The findings of Gracia and Zeballos’ study showed that both groups of consumers and producers (retailers) gained from the implementation of the mandatory traceability systems at the expense of driving up the beef price.

Hobbs *et al.* [19] examined if the implementation of an integrated traceability system would have any impact on consumers’ decisions to buy domestically produced beef and pork. The researchers conducted an experimental auction twice: an ex post trace-back system and an ex ante quality assurance system. Hobbs and coworkers’ main objective was to decompose the economic effect of willingness to pay a premium price to purchase beef and pork under three separate categories. These categories were: (i) for a traceability assurance; (ii) for a food safety assurance; and (iii) for an on-farm production method assurance. Hobbs *et al.* [19] concluded that there was not a significant difference between the willingness to pay a premium price for beef and pork and consumers valued quality assurance for beef more than pork only if it was combined with food safety and on-farm production methods.

## 3. Empirical Analysis

As mentioned earlier, the main objective of this article is to examine consumer preferences to choose between food safety and food quality at the time of purchasing certified farm-raised Atlantic salmon in the province of Newfoundland and Labrador, Canada. This objective can be met by measuring consumers’ willing-to-pay a premium price to buy the product. In general, households look for safe foods with high quality. We adapted Haghiri’s model [6] using a contingent valuation method through specifying a probit regression model, *i.e.*, Equation (1), and estimated the parameters of the model using the maximum likelihood approach whose estimates are consistent, globally concave, and asymptotically efficient [30]. The sample data were comprised of 176 consumers who were residing in the province of Newfoundland and Labrador at the time of survey, of whom 120 were successfully interviewed (a 68.2 percent response rate). The participants in the survey were randomly selected from the provincial telephone directory on the grounds of four lucid geographical areas: east, central, west, and the Labrador region. This method of survey was also used by the provincial government in its field survey [6,16]. The interviews were carried out at different times on weekdays. The consumer survey was comprised of questions related to demographic variables, socioeconomic characteristics, and attitudinal variables. The latter factor, also known as psychographic variables, included consumer attributes related to values, personality, attitudes, interests, beliefs, and lifestyles. We used a 5-point Likert scale to classify consumers’ attitudes towards traceability systems where 1 indicates a minimum level of agreement and 5 shows the maximum level of agreement. The questionnaire also provided information related to where respondents often ate salmon, types of salmon they preferred, their level of consumption, and their knowledge on PCBs.

A summary of the descriptive analysis of the collected data is shown in Table 1. Of the 120 respondents, almost 59 percent were female, 24.2 percent were the secondary salmon buyers in their households, and 29.2 percent were single. It is noticed from Table 1 that 55 percent of the respondents were less than 50 years old, of which 46.9 percent were under the age of 35. Collectively, 58.3 percent of respondents did not have a university degree and almost 48 percent were not employed at the time of survey. Of the 120 respondents, 40 percent had annual household incomes of above CAD 50,000 and 39.6 percent of these households earned more than CAD 80,000. The analysis of the sample data indicated that 34 respondents (28.3 percent) declared an annual household income of less than CAD 30,000. In total, only 25 percent of the respondents inclined to consume farmed Atlantic salmon instead of the wild-caught one because the provincial DFA imposed a quota system on the number of wild-caught salmon to maintain the balance of salmon population. The law also applies to both self-consumption and sports-fishing anglers in the province (6:1097). The results of the survey revealed that respondents had immense knowledge about PCBs. Of the 120 participants in the survey, 69.1 percent were familiar with its adverse impact on human lives [16]. Equation (1) shows the probit regression model in which the dependent variable was defined as whether or not respondents were willing to pay a 15 percent premium price to purchase certified farmed Atlantic salmon (*i.e.*, a simple dichotomous yes/no variable). The amount of the premium price was chosen from the survey as more than two-thirds of respondents declared that they were willing to pay 15 percent more to purchase farm-raised Atlantic salmon that passed through a traceability system.

(1) $$\begin{array}{ccc} WTP_{certifiedproducts} & = & \gamma_{0}+\gamma_{1}\;gen+\gamma_{2}\;age2+\gamma_{3}\;age3+\gamma_{4}\;age4+\gamma_{5}\;marit+\gamma_{6}\;fsz+\\ & & \gamma_{7}\;edu2+\gamma_{8}\;edu3+\gamma_{9}\;inc2+\gamma_{10}\;inc3+\gamma_{11}\;inc4+\gamma_{12}\;saltyp+\\ & & \gamma_{13}\;salpre+\gamma_{14}\;pubknow+\gamma_{15}\;primbuyer+\gamma_{16}\;visfishmkt+\\ & & \gamma_{17}\;readlabel+\gamma_{18}\;pcbknow+\gamma_{19}\;nutri2+\gamma_{20}\;nutri3+\\ & & \gamma_{21}\;nutri4+\varepsilon_{i} \end{array}$$

It is worth mentioning that our study is different from Haghiri’s research as the concept of food quality was not part of the former study [6]. We briefly present the estimation results of the regression model and refer interested readers to an in-depth discussion in Haghiri [6]. Similar to other studies where the contingent valuation method was used, some limitations, such as the incentive compatibility and the existence of hypothetical bias, are applied to this research [19]. 

Table 2 shows the estimated parameters of the regression model. In total, the null hypothesis that expressed all slope coefficients of the model were zero was rejected with 95 percent confidence since the magnitude of the likelihood ratio test was approximately equal to 58.20. The estimated parameter for seniors (AGE4**)** was positive, statistically significant at the 0.05 level, and showed that seniors were 19.7 percent (*p*-value 0.039) more likely to pay a 15 percent premium price to purchase certified farmed Atlantic salmon than those respondents who were less than 35 years old (AGE1, *i.e.*, the control group). The results of the regression model also showed that there was a positive correlation between annual household income and willingness to pay the 15 percent premium price. Coefficients for both higher income brackets, *i.e.*, INC3 and INC4, were positive and statistically significant at the 0.05 and 0.01 level, respectively. Those participants in the survey whose annual household income was above CAD 50,000 were 22.9 percent (*p*-value 0.043) more likely to pay the premium price when compared to those respondents whose annual income was less than CAD 30,000, while those respondents whose annual household income was more than CAD 80,000 were 25.5 percent (*p*-value 0.001) more likely to pay the 15 percent premium price when compared to the control group (households with the income of less than CAD 30,000 per annum). The dummy variable indicating whether respondents preferred either fresh or frozen salmon (SALPRE) was also positive and statistically significant at the 0.01 level. The estimated parameter of SALPRE was 0.4450, implying that these respondents were 44.5 percent (*p*-value 0.010) more likely to pay the premium price to consume certified farmed Atlantic salmon than of those respondents who preferred frozen salmon. As Haghiri [12] stressed, improvements in public knowledge usually have positive impact in their decision-making process at the time of buying certified farm-raised Atlantic salmon. Table 2 shows that those participants in the survey who knew the difference between fresh and wild salmon (PUBKNOW) were, on average, 25.3 percent (*p*-value 0.027) less likely to pay the 15 percent premium price to purchase certified farm-raised Atlantic salmon. Finally, the last independent variable with a statistically significant coefficient at the 0.01 level was the one representing respondents with a weekly salmon consumption of between two and four pounds at the household level (NUTRI3). These respondents were, on average, 27.9 percent (*p*-value 0.001) more likely to pay a 15 percent premium price to purchase certified farmed Atlantic salmon than those respondents who fell in the control group (*i.e.*, households with less than one pound of salmon consumption per week).

As mentioned in the Introduction Section, food quality and food safety have two separate meanings, though they support each other in the same direction to help consumers make their informed decisions. Thus far, we have discussed the matter of food safety. Herein, we review the food quality concept and provide some remarkable findings from the results of the study. Once again, we emphasize that the concept of food quality involves consumer attributes. These attributes have positive impacts on consumers, leading them to have different values for each product in the market. We took into account some consumer attributes, such as originality, freshness, colour, flavour, texture, taste, nutritional value, the animal welfare, and sustainable farming practices in the survey to examine what factors affected respondents’ decisions at the time of purchasing certified farm-raised Atlantic salmon. The result of the survey showed that more than 93 percent of the participants in the survey like the taste of salmon in comparison to other fish species because they believed that salmon has delicate texture when it is cooked. This group of participants in the survey, hypothetically, were amongst those who were most likely to pay the 15 percent premium price to purchase certified farm-raised Atlantic salmon. It is also found that more than 82.5 percent of respondents preferred to eat salmon at home rather than at restaurants, as they could control the flavour when they cooked the fish. Nutritional value is another factor that affects food quality. The survey found that more than 86 percent of respondents preferred to consume fresh Atlantic salmon instead of frozen salmon because there is more nutritional value in fresh salmon. When purchasing salmon, its colour is also a factor that has positive impact on consumers’ decisions. It is shown from the survey that 67.5 percent of respondents declared that the colour of fish is very important to them and this would raise the likelihood of paying a premium price for certified farm-raised Atlantic salmon. Another factor that would affect consumers’ decisions is the origin of the product. The consumption of locally grown foods has recently been the centre of controversial debate amongst stakeholders in the food industry. As Haghiri [31] pointed out, possible outcomes of shifting consumer demand towards local foods is the reduction in consumption of fuel needed to deliver foods, mitigating the amount of greenhouse gas emissions, and reducing global warming, which improves the sustainability of agriculture and aquaculture practicing. Finally, the primal analysis of the survey showed that Newfoundlanders and Labradoreans placed great value on locally produced farm-raised Atlantic salmon. More than 95 percent of respondents in the survey declared that they would prefer to buy certified farm-raised Atlantic salmon if it were produced in Newfoundland and Labrador. The participants in the survey placed lower values on those products that were either imported from the Maritimes provinces or brought from the rest of the country, which, to some extent, implied how important factors such as the place of origin and sustainable farming practices were to the residents of Newfoundland and Labrador.

## 4. Conclusions and Implications for Managers

When the issues of food quality and food safety matter, consumers have no doubt that they will have to pay a premium price to purchase safe and high quality products. In agricultural global markets, the above two concepts usually move together to strengthen consumers’ demand. This article examines a case study on consumer choice between food safety and food quality for farm—raised Atlantic salmon in the province of Newfoundland and Labrador, Canada. After the food incidence of polychlorinated biphenyls in farmed Atlantic salmon in the 2000s, the global market observed a drastic change in the demand for the product. With several policies in place, the global demand for farm-raised Atlantic salmon has rebounded at the expense of increasing demand for high-quality and safe foods. Amongst the suggested policies, we recommend the use of the integration traceability methods and quality control system to restore consumer confidence in the safety of farm-raised Atlantic salmon and, as a result, the market stability. As Magera and Beaton [32] mentioned, the integration traceability methods and quality control system is an internal-and-external traceability system used by most stakeholders in the global food industries. It consists of three distinct, but related, methods, known as the GlobalGAP and Quality Management Program, the Hazard Analysis and Critical Control Points, and the radio frequency identification and quick response code—systems that could, collectively, help consumers have access to detailed information about the foods they consume.

Traceability systems will have positive impact on the economic values of firms, especially when it is associated with food quality, though the outcome may vary from one industry to another [28]. The result of this research showed that age, level of income, freshness, public knowledge about polychlorinated biphenyls, and the frequency of salmon consumption would have positive impact on households’ decisions to consume certified farm-raised Atlantic salmon. In addition, the findings of this study showed that when food quality attributes, such as origin, freshness, colour, flavour, texture, taste, nutritional value, and sustainable farming practices, were combined with the food safety concept the likelihood of paying a premium price to purchase the certified product increases. It is noteworthy to mention that the results of this research should be interpreted cautiously because these findings have been drawn only from the sample data, which, as mentioned earlier, is exposed to common sampling conditions including the existence of hypothetical bias and/or incentive compatibility [33]. That being said, since 67 percent of total provincial aquaculture production and 85 percent of total aquaculture exports are operated by private firms [3], the above impediments can be abated [6]. 

These findings provide some directions for researchers and managers in the global food markets, especially the farmed salmon industry. First, the implementation of traceability systems will bring benefits to both consumers and producers. When the traceability system is placed, consumers receive benefits when food problems arise because the system is able to detect the cause of the food problem. Thus, we suggest managers and market-promoters to highlight this aspect of implementing the traceability system. On the other side, producers, fish-plant processors, and retailers also gain from putting the traceability system in place when food safety incidents occur. Moreover, the income elasticity of demand is generally positive and relatively low for agricultural products (such as farm-raised Atlantic salmon), as opposed to the industrial ones, which means agricultural producers are much more vulnerable against any reduction in demand and/or output prices. Once a traceability system is in place, they are protected from any food crises since they can act immediately to find the cause of the food incident. Besides, proper marketing strategies that focus on the safety of certified farm-raised Atlantic salmon would possibly keep its demand steady. Second, precedent found three distinct groups of consumer attitudes (*i.e.*, “knowledge cognizance”, “price conscious”, and “self-confident”) toward the implementation of traceability system in the salmon industry in Newfoundland and Labrador, Canada [16]. From the managerial perspective, we suggest that the first two consumer segments should be given more attention by marketers as both groups are willing to accept a premium price for certified farmed Atlantic salmon yet their willingness to pay a premium price for each group is different. Therefore, we suggest a new research that identifies the disparity between willingness to accept and willingness to pay a premium price between the two consumers segments to purchase certified farm-raised Atlantic salmon. Third, improving public knowledge about PCBs and its adverse impact on consumers’ health is another angle that managers are urged to concentrate on to encourage households to purchase certified farm-raised Atlantic salmon that has undergone the rigorous GlobalGAP and Quality Management Program (QMP) plan, and has passed through various stages of traceability and quality control systems, such as the Hazard Analysis and Critical Control Points (HACCP) mechanism for the purpose of residue testing. Fourth, the implementation of the traceability system may eventually lead to a shift in the demand for farmed Atlantic salmon through changing consumers’ tastes and preferences. Having the knowledge of the percentage change in the demand will help managers offer a variety of pricing policies to quantify the change in demand. Fifth, it is expected that the traceability system will increase the product price. We propose original studies that explore the possible establishment and function of new economic institutions, such as intermediary firms for marketing certified farm-raised Atlantic salmon. Spulber [34] defined intermediary firms as economic institutions that create and manage markets by acting as intermediaries between consumers and producers. The functionality of such new institutional entities has not been studied in the literature for the salmon industry.

## Figures and Tables

**Table 1:** Summary statistics for the independent variables *.

Variable Name	Frequency	Mean	S.D.
***Gender***			
Female	71	0.5917	0.4936
Male ^*^	49	0.4083	0.4936
***Age***			
Less than 35 years of age ^#^	31	0.2583	0.4396
35–50 years of age	35	0.2917	0.4564
51–65 years of age	29	0.2417	0.4299
More than 65 years of age	25	0.2083	0.4078
***Marital Status***			
Singles ^#^	35	0.2917	0.4564
Married	85	0.7083	0.4564
***Family Size***	120	2.4467	1.2362
***Education***			
High school or less ^#^	60	0.5000	0.5021
Some college	10	0.0833	0.2775
University degree	50	0.4167	0.4951
***Employment Status***			
Employed	62	0.5167	0.5018
Unemployed	22	0.1833	0.3886
Retired	36	0.3000	0.4602
***Annual Household Income***			
Less than $29,999 ^#^	34	0.2833	0.4525
$30,000–$49,999	38	0.3167	0.4671
$50,000–$79,999	29	0.2417	0.4299
$80,000 or more	19	0.1583	0.3666
***Salmon Type Consumption***			
Wild-Caught	90	0.7500	0.4348
Farm-Raised ^#^	30	0.2500	0.4348
***Salmon Type Preference***			
Fresh	104	0.8667	0.3414
Frozen ^#^	16	0.1333	0.3414
***Public Knowledge-Does fresh salmon mean wild salmon?***			
Yes ^#^	49	0.4083	0.4936
No	71	0.5917	0.4936
***Primary Salmon Buyer***			
Yes ^#^	91	0.7583	0.4299
No	29	0.2417	0.4299
***Visiting Fish Market***			
Yes ^#^	51	0.4250	0.4964
No	69	0.5750	0.4964
***Read Salmon Label***			
Yes ^#^	64	0.5333	0.5010
No	56	0.4667	0.5010
***Public Knowledge—Do you know what Polychlorinated biphenyls are?***			
Yes ^#^	83	0.6917	0.4637
No	37	0.3083	0.4637
***Weekly Salmon Consumption***			
Less than 1 pound ^#^	36	0.3000	0.4602
1–2 pounds	50	0.4167	0.4951
2–4 pounds	27	0.2250	0.4193
More than 4 pounds	7	0.0583	0.2354
***Location***			
East	50	0.4167	0.4951
Central	12	0.1000	0.3013
West	45	0.3750	0.4862
Labrador	13	0.1083	0.3121

* S.D., standard deviation. ^#^ The group-category explanatory variable omitted from the regression model to avoid the problem of perfect collinearity. Source: Sample data. See also Haghiri [6] and Haghiri and Simchi [16].

**Table 2:** Estimated Coefficients.

Variable Name	Estimate (*p*-Value)	Change in Probability (*p*-Value)
Constant	−2.5398 (0.024)	-
GEN	−0.3338 (0.500)	−0.0849 (0.490)
AGE2	−0.1593 (0.765)	−0.0430 (0.772)
AGE3	0.7855 (0.237)	0.1684 (0.123)
AGE4 **	1.0122 (0.155)	0.1972 (0.039)
MARIT *	1.0488 (0.054)	0.3171 (0.082)
FMSZ	−0.0927 (0.764)	−0.0243 (0.766)
EDU2	0.3270 (0.656)	0.0747 (0.603)
EDU3	−1.0099 (0.171)	−0.2847 (0.189)
INC2	−0.1735 (0.723)	−0.0467 (0.728)
INC3 **	1.1851 (0.119)	0.2295 (0.043)
INC4 ***	1.7794 (0.058)	0.2548 (0.001)
SALTYP *	−0.8086 (0.140)	−0.1735 (0.062)
SALPRE ***	1.2992 (0.008)	0.4450 (0.010)
PUBKNOW **	−0.9085 (0.023)	−0.2533 (0.027)
PRIMBUYER *	−0.9670 (0.68)	−0.3022 (0.098)
VISFISHMKT	0.5038 (0.330)	0.1273 (0.309)
READLABEL *	0.9243 (0.078)	0.2470 (0.076)
PCBKNOW	0.7043 (0.140)	0.2053 (0.162)
NUTRI2	0.3927 (0.347)	0.0997 (0.341)
NUTRI3 ***	1.6560 (0.016)	0.2792 (0.001)
NUTRI4	0.8768 (0.511)	0.1526 (0.274)
Number of observations	120	
McFadden R-squared (Pseudo R-squared)	0.4014	
Likelihood ratio statistic	58.15	
Degrees of freedom	21	
Prob (ChiSqd > value)	0.0000	

* Significant at 0.10; ** Significant at 0.05; *** Significant at 0.01; Source: Sample data. Adapted from Haghiri [6].
